# Event dependence in U.S. executions

**DOI:** 10.1371/journal.pone.0190244

**Published:** 2018-01-02

**Authors:** Frank R. Baumgartner, Janet M. Box-Steffensmeier, Benjamin W. Campbell

**Affiliations:** 1 Department of Political Science, UNC-Chapel Hill, 313 Hamilton Hall, Chapel Hill, NC, 27599-3265, United States of America; 2 Department of Political Science, The Ohio State University, 230 North Oval Mall, Columbus, OH 43210, United States of America; Northwestern University, UNITED STATES

## Abstract

Since 1976, the United States has seen over 1,400 judicial executions, and these have been highly concentrated in only a few states and counties. The number of executions across counties appears to fit a stretched distribution. These distributions are typically reflective of self-reinforcing processes where the probability of observing an event increases for each previous event. To examine these processes, we employ two-pronged empirical strategy. First, we utilize bootstrapped Kolmogorov-Smirnov tests to determine whether the pattern of executions reflect a stretched distribution, and confirm that they do. Second, we test for event-dependence using the Conditional Frailty Model. Our tests estimate the monthly hazard of an execution in a given county, accounting for the number of previous executions, homicides, poverty, and population demographics. Controlling for other factors, we find that the number of prior executions in a county increases the probability of the next execution and accelerates its timing. Once a jurisdiction goes down a given path, the path becomes self-reinforcing, causing the counties to separate out into those never executing (the vast majority of counties) and those which use the punishment frequently. This finding is of great legal and normative concern, and ultimately, may not be consistent with the equal protection clause of the U.S. Constitution.

## Introduction

**Fig 1:**
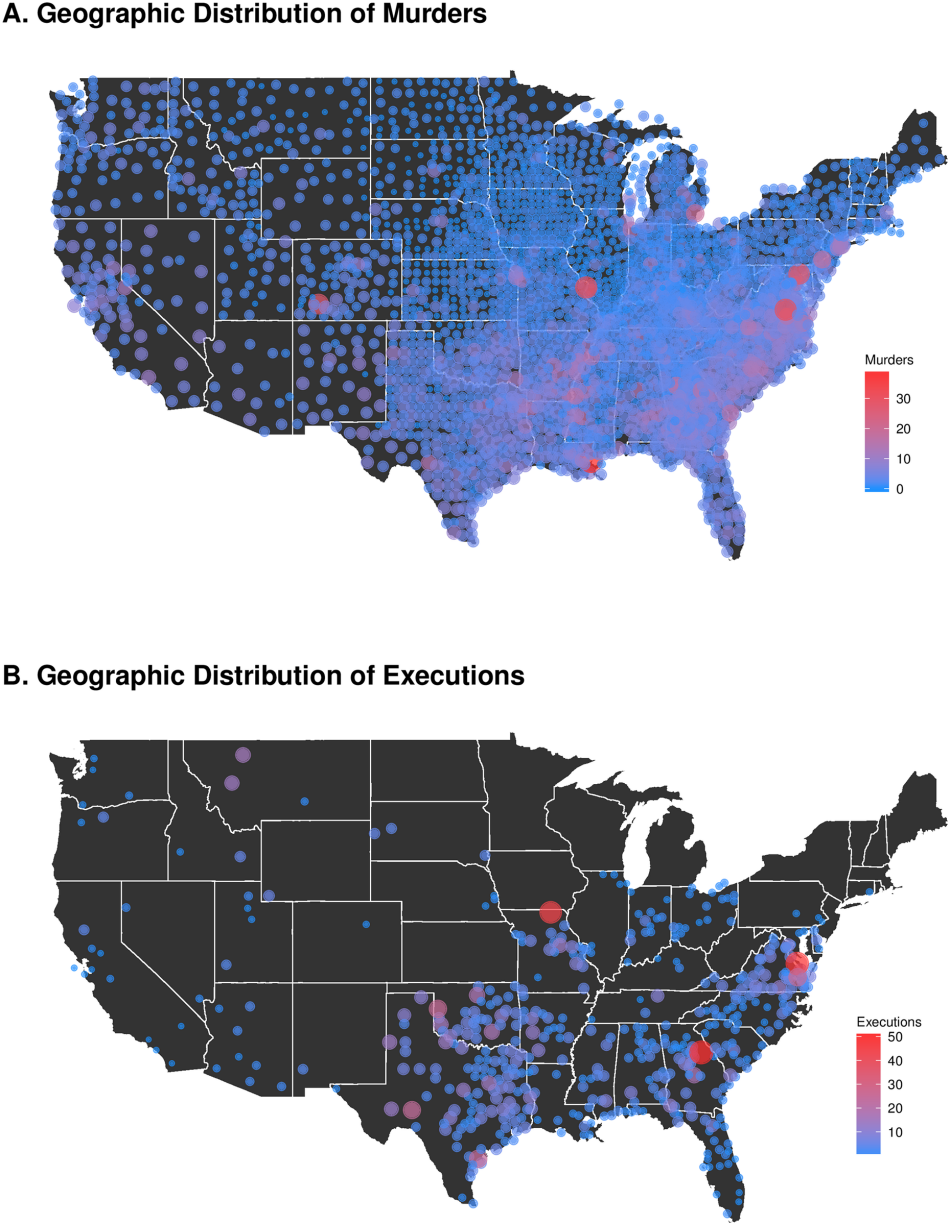
Average murders and executions by county per year per 100,000 residents, 1977–2014. Maps present the average number of murders and executions experienced per year per 100,000 residents by each county between 1977 and 2014.

The U.S. Supreme Court validated the modern death penalty in its *Gregg v. Georgia* decision in 1976. Since then, over 1,400 judicial executions have followed, through the end of 2015. These executions have been highly concentrated in a small number of jurisdictions, however: Texas had 513 where the next closest state (Oklahoma) had just 112, and the average across all death penalty states is fewer than ten. Across the 3,000+ counties in the U.S., the vast majority have executed not a single prisoner, whereas Harris County, Texas (home to Houston) has executed 125. Just twenty U.S. counties have executed 10 or more individuals in the 40 years since the *Gregg* decision. Here, we refer to the county where the death sentence was handed down. County-level prosecutors make the decision of whether to seek death, and trials are typically in the county of the crime. Therefore, the county is of interest even through states, not counties, typically carry out the executions. Fig 1A shows the concentration of murders; Fig 1B presents an equivalent presentation of executions.

Fig 1 makes two things apparent: executions are highly concentrated, and the counties with the greatest number of executions are not the same as those with the homicides, controlling for population size. New Orleans has the greatest number of homicides per year per 100,000 residents, with Washington DC, St. Louis, Richmond, and Baltimore following. Wayne County, Michigan (home to Detroit) is the only one of the high-homicide locations in a state without the death penalty. This poses an interesting puzzle that runs counter to the prior expectations of many [1–4]—if murders and the opportunity for utilizing the death penalty appear to be unrelated, *what explains this clustering of executions?* This question motivates our research. We hold that executions reflect a self-reinforcing process. We present empirical support for this theory through a series of analyses that demonstrate that executions reflect a power law or log normal self-reinforcing process. We do so by utilizing bootstrapped Kolmogorov-Smirnov tests [5] and further use conditional frailty models to test for event dependence [6, 7]. Our finding that executions are conditional on the number of previous executions both explains their high concentration in just a few jurisdictions and raises important substantive concerns. After all, the historical track record of a given county in carrying out previous death sentences should not be a “legally relevant factor” in determining whether the next inmate deserves the ultimate punishment. That should relate solely to the nature of the crime and the characteristics of the offender.

## Correlated v. random local variation

As described in [8], with further analyses available in [9–11] no single actor determines whether an execution will take place. Homicides occur with great regularity across the US, some of which meet the statutory requirements to be eligible for a capital prosecution. Prosecutors may vary in their proclivities to seek death. Capital defenders, typically part of a public defender’s office or appointed by the Court, may be more or less well funded, active, and successful. Juries, judges and appellate courts may be more or less supportive of the death penalty. While some degree of local variation is to be expected because of random fluctuations in any variable, if the many actors involved in capital punishment showed independent random fluctuation, the Central Limit Theorem would dictate a Gaussian distribution of outcomes. If, on the other hand, variation is correlated, then, as [10] explains, such a process “tends to produce momentum in favor or against sustained capital activity”, producing “a separating equilibrium for death penalties and executions: a few capitally active localities, and many more than tend towards abstention” (p. 264). If a prosecutor bases their decision to seek death on the basis of previous experience in the county and an assessment of the likelihood that other actors will go along with the decision, and other actors do the same, then event dependency can ensue. The same crime, in two different counties (or at two different times) would have dramatically different odds of leading to execution. Our test is designed to capture these dynamics, which is exactly what we uncover.

## Materials and methods

To demonstrate support for the proposition that judicial executions reflect a self-reinforcing processes, we adopt a two-pronged modeling approach. The first prong involves using bootstrapped Kolmogorov-Smirnov (KS) tests to infer the functional form of the frequency distribution of executions across counties. Should the distribution of executions follow a stretched distribution, such as the power law or exponential, there would be suggestive evidence of event dependence [5]. The second prong includes the use of survival analysis, and in particular the conditional frailty model [6, 7], to infer whether this self-reinforcing process holds once accounting for relevant confounding variables. All analyses were conducted using the R Statistical Computing Environment. All code and source data files for reproduction will be made available publicly through the Harvard Dataverse and the Interuniversity Consortium for Political and Social Research (ICPSR) data repository. In this section, we describe the data used for analyses and this two-pronged modeling approach.

### Statistical analyses

#### Bootstrapped Kolmogorov-Smirnov (KS) tests to infer distributional form

To infer the functional form of a particular set of outcomes, [5] recommend the use of bootstrapped Kolmogorov-Smirnov (KS) tests. This strategy allows one to compare the distribution of observed data to data simulated from a distribution with parameters determined by the observed data. These two distributions are then compared using KS tests. Should the test yield a p-value greater than *α* = 0.05, then the null that the two distributions are of the same functional form cannot be rejected. We conduct four tests to determine the distributional form that best fits the observed data—power law, log normal, Poisson, or exponential—with 100,000 bootstrap replications per form. Each of these distributions, save the Poisson, is referred to a “stretched” distribution and is reflective of a self-reinforcing process whereby the probability of observing an event increases for each previous observation of an event. As such, we would expect bootstrapped KS test p-values greater than *α* = 0.05 for the power law, log normal, and exponential distributions and p-values less than *α* = 0.05 for the Poisson.

#### Using the conditional frailty model to test for event dependence

While bootstrapped KS tests are useful in providing suggestive evidence that phenomena appear to reflect a self-reinforcing process, they cannot account for observed or unobserved confounding variables that may produce spurious evidence of event dependence. To account for these confounding variables our model of choice, we use the the conditional frailty model. This model can be understood as a special case of the Cox proportional hazard model. The conditional frailty model allows scholars to explicitly account for unobserved unit-level heterogeneity, while also incorporating event dependence that may pose inferential challenges [6, 7]. These extensions allow for a model that minimizes bias while increasing efficiency relative to alternative models that may be used to test theories of event dependence [6, 7]. In addition, the conditional frailty model performs better under conditions of event dependence and heterogeneity, making this model the safest choice [6, 7].

The model presented in the manuscript includes the variables described in the next subsection in addition to a state-level frailty term to account for any unobserved heterogeneity at the state level. This term is said to increase estimation efficiency while accounting for any confounding effects that may exist at the state level, including, for example, a culture tolerant of the death penalty. This frailty term is estimated using the gamma distribution with variance estimated according to an expectation-maximization algorithm.

### Data

The execution data used for both analyses draw from data developed by [9]. This data consists of all 1,422 US executions measured across 474 counties in 34 different states from 1977 to 2014, containing information on the date of execution and the county of conviction. The list of executions was originally sourced from the widely available “Espy file” of known executions [12], confirmed and supplemented with further follow up by [9]. This provides the distribution of executions that is examined through both analyses. For the conditional frailty model presented in the manuscript, observations are modeled as the gap time between executions with three levels of event dependence stratification, the number of homicides in the county, racial threat in the county, poverty rate, and the population of the county. To clarify, the county-unit of analysis refers to the county in which a sentence was handed down, not the county in which the execution takes place. Executions are typically carried out in a single location in each state, but the legal actors involved relate to the county of conviction or to the state capital, not the location of the execution chamber. The homicides control variable is sourced from [13], who provide the number of homicides per year, by county. This was compiled from annual Supplemental Homicide Reports (that is, FBI data). The inclusion of this control is essential, as it seems intuitive that the number of executions that a county experiences may be correlated with the number of homicides [13]. The second control, racial threat, is an important control as theory tells us that as the members of a minority group increase to a certain point, the majority race will seek to impose higher levels of social control. As such, the death penalty represents one such example of a punitive measure for law breaking. This variable is coded according to the conventionally used routine: 100 − |70−*percentage of population white*| with data sourced from the census [14]. A similar story could be told of poverty—the amount of poverty in a county may drive executions and the overall prevalence of murder. This variable is coded as the percentage of the population of the county that is in poverty, with data sourced from the census. The final control included, the population of a county, could be said to influence executions as counties with large populations may be more likely to experience large numbers of murder, and as a function of prosecutions, more executions. Large counties also have larger and more professionalized district attorney’s offices. Data for this variable is taken from the census.

## Results

**Fig 2:**
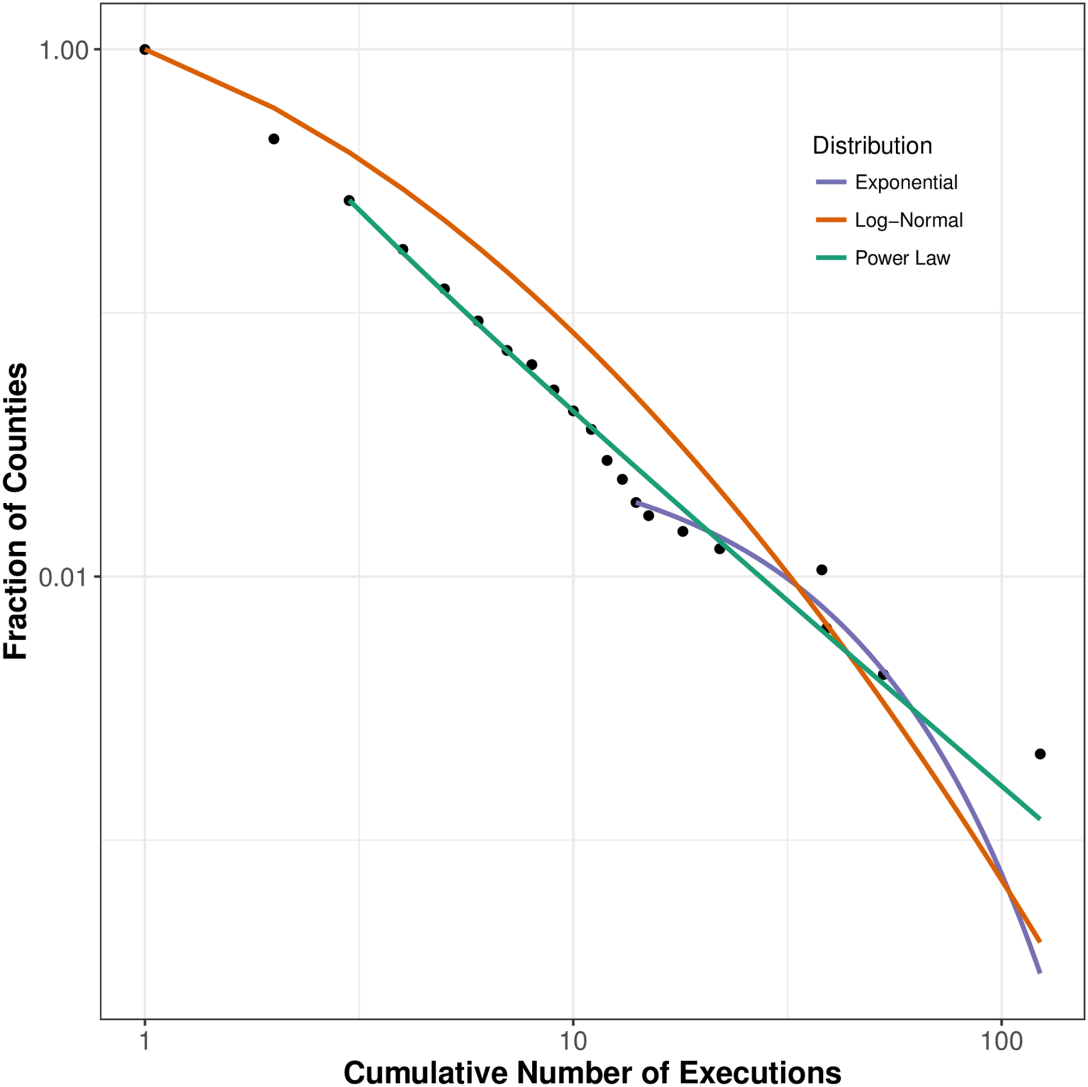
Log-log plot of executions by county. Each colored line represents a distribution fitted to the observed data.

We start by presenting naive evidence suggesting support for our hypothesis in Fig 2, which demonstrates that the concentration of executions appears to approximate a power law or log-normal distribution. That is to say, the distribution appears to be linear or approaching linearity on a log-log scale. The high concentration of executions in such a small number of judicial jurisdictions can be observed whether looking across the 50 states, across the counties as done in Fig 2, within the largest executing states (e.g., across the counties of within Texas or Oklahoma, for example), or across countries of the world. These patterns have already been alluded to by the senior author in other publications [8, 9]. However, this evidence is highly descriptive and does not rule out the potential for these data to be distributed according to another non-stretched distribution, such as the Poisson, or confounded by the population of or murders within a county. Thus, this paper asks not just about the patterns of these executions, but the substantive meaning of these patterns and the relative robustness of the pattern uncovered by [8]. We turn to this question in the following sections.

### Distributional form of death penalty executions

It is often suggested that the distribution of many events appear to fit a power law (or another stretched) distribution on the log-log scale, and that such visual checks are not confirmatory evidence [5]. We agree with this criticism, and to provide additional evidence, we utilize bootstrapped Kolmogorov-Smirnov tests using the routine described by [5] to rule out the possibility that the distribution of executions does not appear to be be distributed according to a power law or some other stretched distribution like the log-normal or exponential. Each of these tests were conducted on a bootstrapped sample which included 100,000 replications. Results from these analyses are presented in Table 1.

**Table 1:** Comparison of the fitted distributions for executions.

Distribution	Bootstrapped K-S *p*
Power Law	0.79
Log Normal	0.83
Poisson	0.00
Exponential	0.37

Overall, there appears to be significant support for our proposition. The distribution of executions is distributed according to some self-reinforcing process. This is demonstrated by the support for the power law, log normal, and exponential, and the lack of support for the Poisson. This leads us to conclude that the data appear to be distributed either exponential, log normal, power law. Any of these stretched distributions reflects a variation on a theme—a self-reinforcing and fat-tailed distribution. This is naive support for our proposition of event dependence in death penalty executions. These tests, however, cannot account for the population of a county, or the number of murders it experiences, which may influence its number of executions. To resolve this concern, we move towards a more proper identification strategy in the following section.

### Identifying event dependence in executions

Given that we know the distribution of executions follows a stretched distribution, the question is can we demonstrate that a self-reinforcing dynamic produced that empirical distribution even after accounting for relevant confounders? Our empirical strategy is to estimate the monthly hazard rate for each county in the United States over the entire modern period of the death penalty, controlling for population size, poverty rate, homicides, “minority threat” [14], and crucially, the number of previous executions the jurisdiction has carried out. This includes 3,607 county-gap time observations, reflecting a census of all relevant cases of counties with the death penalty. We use state-level fixed effects as well in the form of a shared-state frailty term that may account for unobserved state-level confounders. In addition, we exclude counties in states that do not have the death penalty. Nine states abolished the death penalty during the 1977–2015 period, and we exclude counties in those states for the years after abolition.

We are interested in the effects of previous executions on the likelihood of observing a subsequent execution within a fixed time period. But first, let us assess the control variables in the model. Population size and homicides are perhaps obvious: large jurisdictions, and those with many homicides, will naturally give rise to the opportunity for more executions. Given that the distribution of counties in the US is extremely skewed with respect to population, with many counties with small populations and just a few with large populations, we want to ensure that the self-reinforcing aspect of executions cannot be accounted for by these variables; it cannot. We control for poverty and racial dynamics because of the possible effect of poverty on crime rates and for racial threat because of the possible effect of the use of punitive criminal justice sanctions in areas with particular racial dynamics. Crucially, we are not interested in these effects (and indeed, once we control for population, neither homicides nor poverty are significant in the statistical results, and racial threat has only a small bearing). Rather, we need them in the model to assure that our event-dependence modeling be accurate.

Within the context of event history modeling, we assess event dependence by examining the relative hazard functions across different event strata, defined by the number of executions previously carried out. The three strata are clustered as: 0, 1, and 2 or more executions. This clustering routine was chosen to ensure a sufficient number of observations at each stratum to ensure that one or two choice counties did not drive results and lead to Type 1 errors, and is fairly standard across the Event History literature. Models estimated with varying strata revealed similar results. We estimated a fully specified conditional frailty model with a state-level frailty term, event stratification, and the relevant confounding variables including county-level homicides, poverty rate, racial tension, and population [6, 7]. The frailty term accounts for any unobserved heterogeneity across states and thus any unobserved state-level confounders. For example, all counties in the same state would be subject to the same decisions informed by that state’s Supreme Court decisions, Governor’s scheduling decisions, or Corrections Department protocols covering the administration of executions and availability of lethal injection drugs. Similarly, our state-level controls provide assurance that our results cannot be explained by questions of legal precedence. All localities are bound by decisions of the US Supreme Court, but decisions by US Circuit Courts, US District Courts, or state supreme courts would apply only within the relevant jurisdictions. None of these would vary, however, within the boundaries of a given state. Our state-level frailty term is therefore essential to an appropriate modeling strategy accounting for different state laws as well as different legal precedents. While county-level frailty terms may capture the most fine-grained degree of variation that could occur between groups of observations, such terms could not be successfully estimated according to an acceptable convergence criteria.

**Fig 3:**
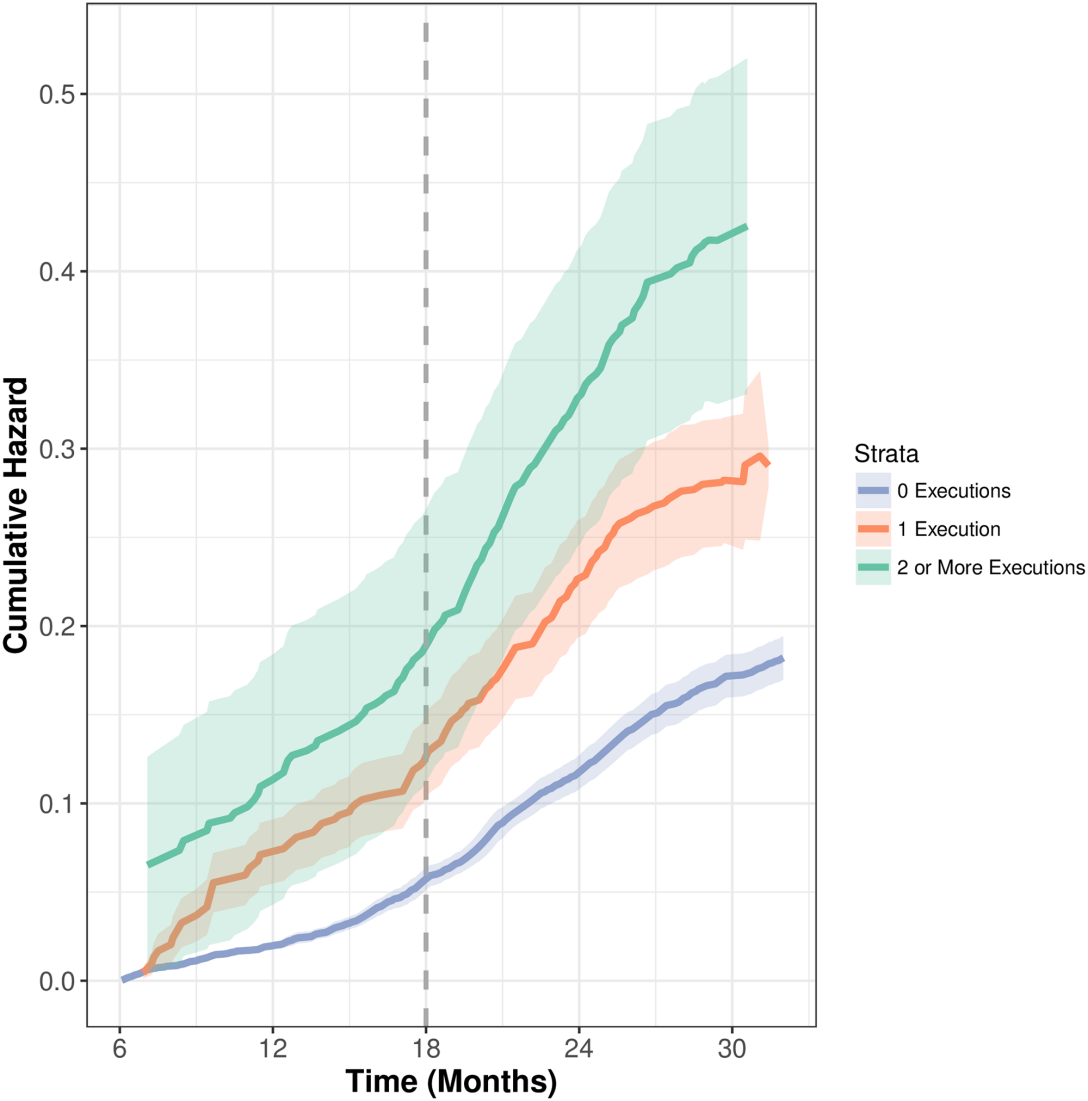
Cumulative hazard functions for fully specified conditional frailty model. Specified with state-level frailty term and three levels of event dependence. 1000 bootstrap replications were used to generate hazards.

To account for sparsity in higher-levels of the strata, we utilize a bootstrapping routine to generate the cumulative hazards presented, but rely upon a non-bootstrapped model for all other discussions. The conditional frailty model estimated according to the specification previously outlined is consistent with theoretical expectations. Fig 3 demonstrates a large amount of event dependence in the frequency of executions as the number of executions in the series increases, even once accounting for the number of murders within the county, or its population. In particular, it demonstrates that as time progresses, counties are increasingly likely to conduct another execution, but that the hazard rate depends crucially on how many previous executions have taken place. The three curves are placed as expected, and become increasingly steep, suggesting more rapid increases in the probability of the next execution for those with more previous executions. In addition, their 95% bootstrapped confidence intervals do not overlap at the higher levels, demonstrating statistical significance of event dependence at the *α* = 0.05 level. Among the counties that have never executed an inmate, the light blue curve in Fig 3, probabilities of execution remain low, regardless of time. While one may expect the never-executing counties to have estimated cumulative hazards of zero, a value greater than zero is expected because these counties have values on the predictor variables whose coefficients are estimated with other strata, and as such, inevitably contribute to the fitted cumulative hazard values. In fact, diagnostics presented in the S1 Appendix demonstrate that the model accounts for these never-executers quite well. The curve for those counties that have one prior execution, the orange curve, increases more rapidly over time and is statistically distinguishable from the baseline curve. This trend continues for the highest-order event dependence (green curve) which becomes statistically distinguishable from all previous strata as time progresses.

A clear substantive interpretation of this is shown at the dashed line representing month 18 in Fig 3 which shows the relative conditional probabilities of observing an execution at 18 months across the full range of strata. At 18 months, the probability of observing an execution for counties in the three groups specified increase from 0.04 to 0.11, to 0.19. In other words, the probability of an execution for a county with previous executions relative to those that have never experienced the executions is approximately five times higher. Note that the model controls for homicides, population, poverty, minority population, and state. Additional model diagnostics and robustness checks are provided in the S1 Appendix.

Of course, some prosecutors seek the death penalty more than others. Some counties are more violent than others. Racial tensions or poverty, combined with crime, may make the local political culture different from place to place. The effects of these controls are presented in Table 2. Only the effect of two variables are robust at any conventional threshold. First, the racial threat variable is significant and in the expected direction. As the white population of a county decreases, which is to say, racial threat increases, there is an increase in the cumulative hazard, or likelihood, of observing an event. This is consistent with previous findings about the enforcement of disproportionate punishments in racially polarized counties and is of particular normative importance. To date, this relationship has not been established for punishments as extreme as the death penalty [15]. This result is exceptionally problematic, it demonstrates that the death penalty may be used in some cases as a means to control communities of color. The same effect holds for the population variable, as the population of a county increases, so does the likelihood of observing another execution. This seems intuitive, and is not particularly novel; as the number of individuals living in a county increases, naively, the number of potential murders and convictions that would occur should increase proportionately. Neither poverty nor homicides produce significant effects. Note that homicides are closely related to population size.

**Table 2:** Conditional frailty model results for controls.

Model 1	
Homicides	−0.00 (0.00)
Percent in Poverty	−0.01 (0.01)
Racial Threat	0.01* (0.00)
ln Population	0.80** (0.04)
AIC	9285.39
BIC	9212.73
R^2^	0.30
Max. R^2^	0.96
Num. events	832
Num. obs.	3136
Missings	471
PH test	0.15

**p < 0.001,

*p < 0.01

The model presented in Table 2 seems to fare quite well with respect to model fit. The inclusion of the frailty term and event dependence maximizes the R-squared of the model to 0.96 from 0.30. Relative to a baseline model that mirrors the model specification in Table 2 excluding event dependence, there is a significant improvement in model fit. Incorporating event dependence, our primary quantity of interest, reduces the Bayesian Information Criterion (BIC) by 15.2% from 10862.33 to 9212.73. This is demonstrative of superior model fit and the added explanatory power of event dependence when considering executions. Unfortunately, further model fit assessments are unavailable for conditional frailty models. In particular, there does not currently exist a cross-validation approach to the conditional frailty model, nor any other out-of-sample model prediction techniques. However, conventional Cox proportional hazards diagnostics are typically considered sufficient to demonstrate model fit. These model diagnostics are presented in the S1 Appendix.

One may note that we do not model dependence in sentencing and as such, are unable to examine dependence between sentences as fewer than 20 percent of death sentences result in an execution, as explained in [9]. Therefore, we cannot examine whether a death sentence increases the likelihood that more cases will be raised and brought to trial by prosecutors seeking the death sentence, but such questions are beyond the scope of this particular paper and are unanswerable by the data currently available. As such, we leave this question open to future inquiry.

## Limitations

It is worth noting that our study is not without its limitations. We have tried our best to acknowledge the relatively dynamic landscape that is the American experience with the death penalty; between 1977 and 2014 there were many changes affecting states in potentially idiosyncratic ways. We have explicitly accounted for changes that would legally preclude a state from using the death penalty. There are, however, a variety of immeasurable changes. These changes might include (but are not limited to) states considering abolition without formally outlawing the death penalty, federal or state moratoria halting executions, and changes in the conditions allowing for death sentences or in the availability of execution drugs. We have attempted to account for these changes by using a conditional frailty model with a state-specific frailty term. This modeling approach should account for these immeasurable factors as they only vary across states, but not within states.

Nevertheless, immeasurable variables driving event dependence that vary within states at the county-level represent a set of limitations. For example, we should note that we do not have data on the number of capital-eligible homicides which would more directly explain a county’s decision to use the death penalty than the number of total homicides experienced. Reliable data on the number of capital-eligible homicides by county is unavailable but ultimately likely to be very highly correlated with our measure of all total homicides.

Finally, we should note that we do not model event dependence in death sentences but rather executions. While modeling sentences is desirable as it is a more direct test of our proposed theoretical mechanism and is less likely to be affected by political decisions or drug shortages, such a study is impossible as data on death sentences are presently unavailable. Our work on dependence in executions is the most comprehensive study to date, and we save an extension to sentencing for future research.

## Discussion

We find evidence of a self-reinforcing mechanism for executions, even when accounting for important covariates such as population, racial threat, homicides, state, or poverty. Previously this has been suspected, only considered through examining naive evidence. As counties have more and more experience with the death penalty, they are increasingly likely to use the death penalty and do so at quicker rates. Not only is this made evident by the different slopes of hazard curves in Fig 3, but the different probabilities of observing an execution at a fixed point in time like 18 months.

The United States Constitution puts crime squarely in the realm of the states, making some degree of local variation inevitable, and this is made stronger by locally elected district attorneys and the powerful role for citizen-based juries. There is no reason, in other words, to expect uniformity in the use of the death penalty across jurisdictions. What we observe, however, is not consistent with a random-fluctuation model. A stretched distribution is consistent only with some form of self-reinforcing mechanism, and in this paper we have tested a model whereby individual jurisdictions adapt to their own histories, over and above such legally or socially relevant factors as changing crime rates, poverty, population, state judicial elections, or even homicides. The extreme concentration of cases in just a few jurisdictions implies that the death penalty is not an option in most American communities. The vast majority have none at all, even across 40 years of experience, and often after thousands of homicides. Rather, the counties separate out into high- and low-execution users based on habit.

A self-reinforcing process suggests that decision-makers may base their actions on their expectations of the actions of others. If prosecutors believe that juries will never support a death sentence, because none ever has, they may decide that seeking it is a poor use of limited resources. If they believe, on the other hand, that local history shows that other actors will carry the process all the way through to completion, they may feel compelled to go along. There is nothing wrong per se in these expectations affecting local decision makers. However, the US Supreme Court has never held that the number of previous executions in a county should be a relevant consideration in deciding who gets death and who lives. This should relate to the nature of the crime and the characteristics of the defendant, according to the Court. Local history should neither be an aggravating nor a mitigating factor in determining who gets death. But it is.

## Supporting information

S1 Appendix Supplementary information appendix. This document contains additional robustness checks and diagnostics associated with the manuscript “Event Dependence in U.S. Executions”. In particular, it contains an assessment of the proportional hazards assumption and residuals (Cox-Snell, Martingale, Deviance) for the presented Conditional Frailty Model. In addition, we assess the possibility that influential observations may influence the results presented. Overall, the Conditional Frailty Model presented appears to fit particularly well and perform well with respect to essential diagnostics.
(PDF)
