# Supplementary material for: Event dependence in U.S. executions
Source: PLoS One. 2018 Jan 2;13(1):e0190244. doi: 10.1371/journal.pone.0190244 (PMC5749737; doi:10.1371/journal.pone.0190244)
Supplement: S1 Appendix — This document contains additional robustness checks and diagnostics associated with the manuscript “Event Dependence in U.S. Executions”. In particular, it contains an assessment of the proportional hazards assumption and residuals (Cox-Snell, Martingale, Deviance) for the presented Conditional Frailty Model. In addition, we assess the possibility that influential observations may influence the results presented. Overall, the Conditional Frailty Model presented appears to fit particularly well and perform well with respect to essential diagnostics. (PDF) [file pone.0190244.s001.pdf]

Supplementary Information (SI) Appendix: Event Dependence in U.S. Executions

Frank R. Baumgartner[1,*,+], **Janet M. Box-Steffensmeier**[2,+], **and Benjamin W. Campbell**[2,+]

[1]Department of Political Science, UNC-Chapel Hill, 313 Hamilton Hall, Chapel Hill, NC 27599-3265, United States
[2]Department of Political Science, The Ohio State University, 230 North Oval Mall, Columbus, Ohio 43210, United States
[*]Frankb@unc.edu
[+]These authors contributed equally to this work.

ABSTRACT

This document contains additional robustness checks and diagnostics associated with the manuscript "Event Dependence in U.S. Executions". In particular, it contains an assessment of the proportional hazards assumption and residuals (Cox-Snell, Martingale, Deviance) for the presented Conditional Frailty Model. In addition, we assess the possibility that influential observations may influence the results presented. Overall, the Conditional Frailty Model presented appears to fit particularly well and perform well with respect to essential diagnostics.

The model presented in the manuscript must be assessed in three ways. First, whether the model violates the proportional hazards assumption, an essential assumption for the identification of the Conditional Frailty Model. Second, whether the model appears problematic from the perspective of any conventional diagnostic, and third, whether we do a poor job predicting counties that have the death penalty but never utilize it. Any of these concerns would be problematic. We examine these in this SI Appendix.

Proportional Hazards

As discussed previously, the last row in Table 2 in the manuscript shows that the model presented in the manuscript passes the global proportional hazards test at the conventional 0.10 threshold. Given this, we feel little reason to hold our results as suspect as a result of violating this assumption.

Cox-Snell Residuals

Figure 1 presents the Cox-Snell Residuals for the model presented in the manuscript. In general, the model appears to fit fairly well. While the residuals across strata do not perfectly hug the unit-exponential line, they do not look particularly problematic.

Martingale Residuals

Figure 2 presents the Martingale Residuals for the full conditional frailty model presented in the manuscript. Ideally, one would want to see residuals that appear fairly linear with a constant distribution of residuals around zero. These residuals appear to be fairly linear, and the slope for each line appears to approximate zero. This does not appear to be the case for Homicides, which is not particularly troubling. There do not appear to be significant differences in the residuals when iteratively removing covariates.

Influential Observations

In an effort to examine the influence certain observations have over model fit, we perform three analyses. First, we examine the change in coefficient estimates from iteratively removing observations. These DFBetas are presented in Figure 3. Overall, it does not appear that any particular observations greatly influence coefficient estimates.

However, given that we are ultimately interested in event dependence, something not captured by covariates, these may not be particularly useful. As such, we estimate the two versions of the full conditional frailty model excluding Harris and Dallas counties – the two counties known for their heavy-handed use of the death penalty. Figures 4 and 5 present the cumulative hazard functions by strata for models excluding Dallas and Harris counties respectively. It is worth noting that upon excluding these observations the confidence intervals become more narrow, which makes sense given that Harris and Dallas may increase the variance by strata. Otherwise, the results do not change much, and in fact, the distinctions between hazard curves become clearer. We also estimate the full conditional frailty model on a sample that excludes any counties that have outlawed the death penalty at any point during the sampling window. In the analysis presented in the manuscript, Rhode Island for example, was included in the sample for 1977 through 1983. It was taken out of the sample as it banned the death penalty in 1984. However, during those seven years, Rhode Island never executed an inmate. This might be a function of a broader state-based dynamic that made the death penalty functionally irrelevant. One might expect these dynamics to play out in similar states, such as Massachusetts or New York. Figure 6 presents the cumulative hazard functions by strata excluding these states. Overall, evidence of event dependence persists even when excluding future abolitionist states; the slope of the hazard function for the first stratum only experiences a marginal increase.

Deviance Observations

The final diagnostic check performed on the presented conditional frailty model is an examination of the deviance residuals for the model. The right-most figure in Figure 7 shows that our initial concern of poorly predicting never-executers does not appear to emerge. In other words, the deviance residuals do not vary significantly according to observation or their overall gap time. This would be evidence that the stratification and state-based frailty term does a good job in accounting for these never-executers.

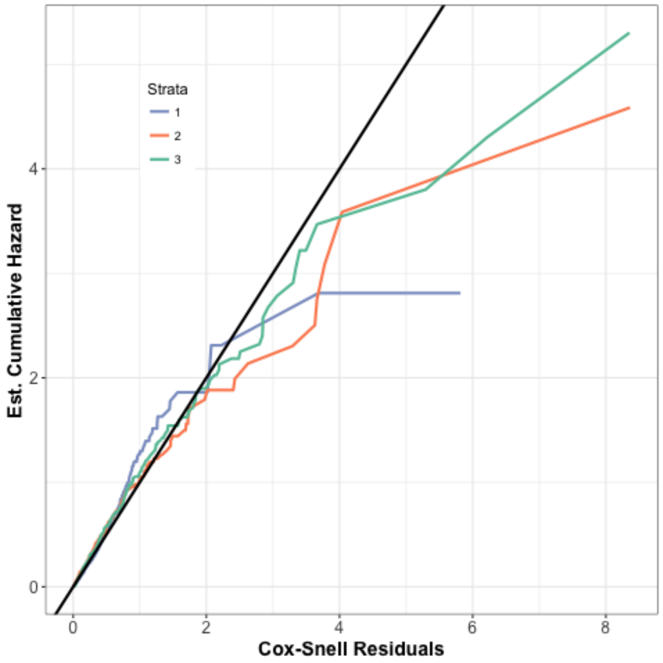

]h!]

Figure 1. Cox-Snell Residuals for fully specified conditional frailty model. Residuals are extracted according to strata..

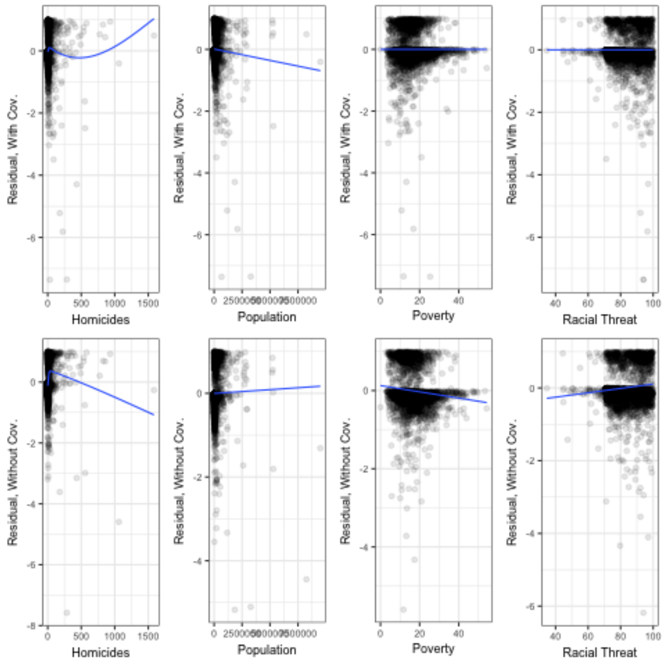

Figure 2. Martingale Residuals for fully specified conditional frailty model. Residuals are extracted with and without the covariate included. The line calculated for population, poverty, and racial threat is fit linear. The homicides variable residuals are fit with a Generalized Additive Model curve.

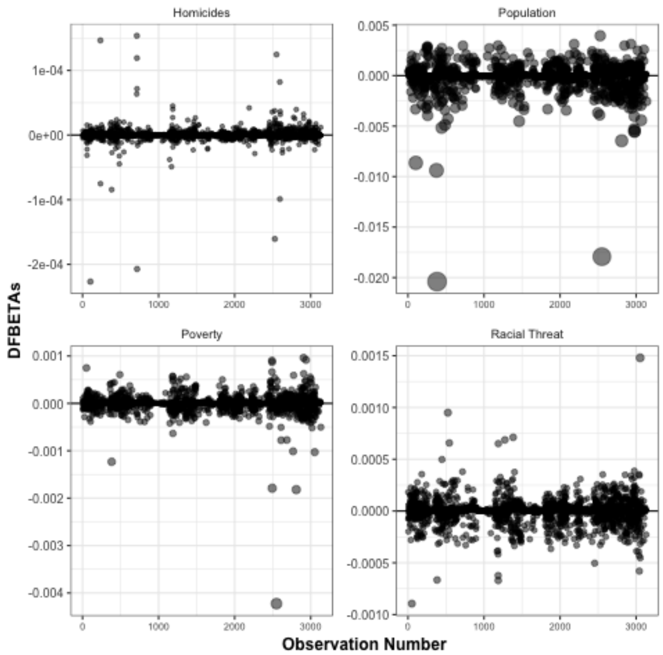

Figure 3. DFBetas for fully specified conditional frailty model. Observations are sized according to relative distance from zero.

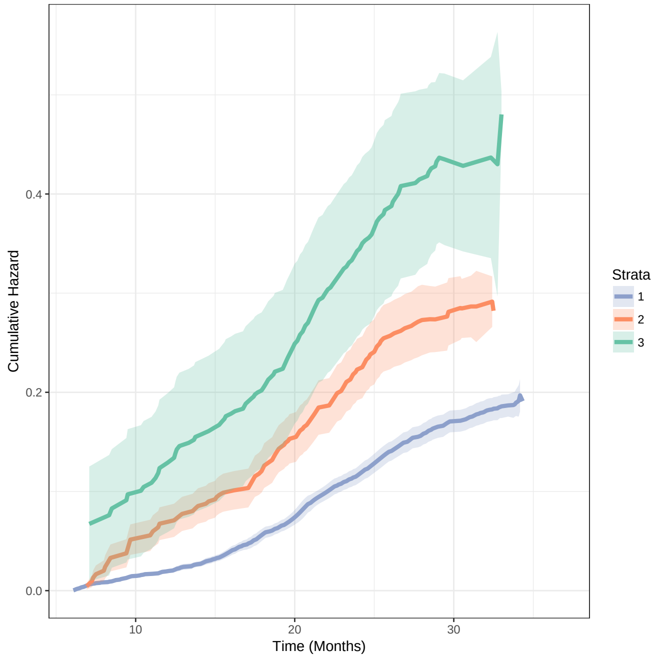

Figure 4. Cumulative hazard curves for fully specified conditional frailty model fit excluding Dallas County. Model specified according to the previously discussed model specifications, the only difference being the exclusion of Dallas County which ranks second to the top with respect to executions. 1000 Bootstrap replications.

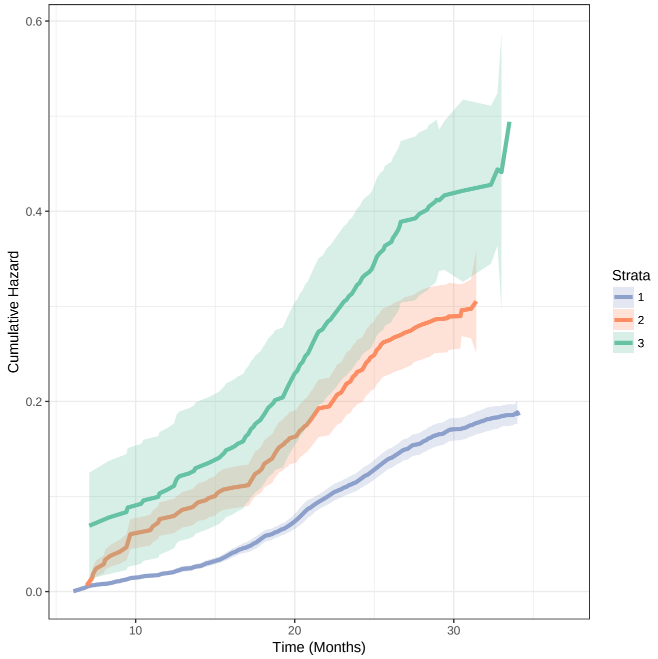

Figure 5. Cumulative hazard curves for fully specified conditional frailty model fit excluding Harris County. Model specified according to the previously discussed model specifications, the only differencing being the exclusion of Harris County which ranks at the top with respect to executions. 1000 bootstrap replications.

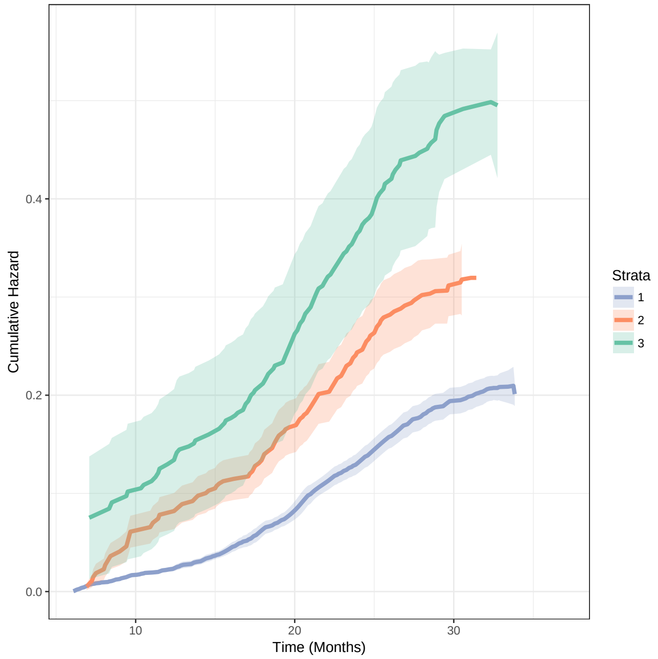

Figure 6. Cumulative hazard curves for fully specified conditional frailty model fit excluding states that have gone on to abolish the death penalty. Model specified according to the previously discussed model specifications, the only difference being the exclusion of any counties in states that have outlawed the death penalty. 1000 bootstrap replications.

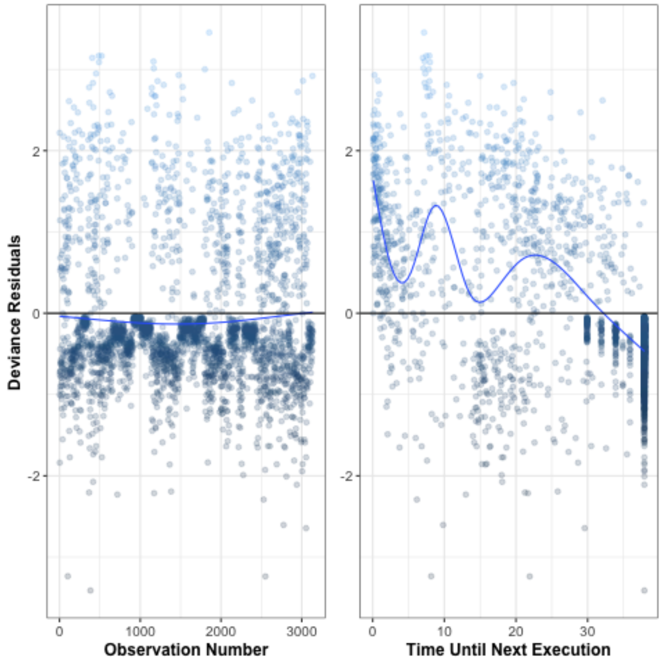

Figure 7. Deviance residuals for fully specified conditional frailty model. Model specified according to the previously discussed model specifications. The left figure shows observations that are poorly predicted according to observation number; the right figure shows this with respect to the time until the next execution.
